# Diffusion-derived parameters in lesions, peri-lesion and normal-appearing white matter in multiple sclerosis using tensor, kurtosis and fixel-based analysis

**DOI:** 10.1177/0271678X221107953

**Published:** 2022-06-25

**Authors:** Chris WJ van der Weijden, Anouk van der Hoorn, Jan Hendrik Potze, Remco J Renken, Ronald JH Borra, Rudi AJO Dierckx, Ingomar W Gutmann, Hakim Ouaalam, Davood Karimi, Ali Gholipour, Simon K Warfield, Erik FJ de Vries, Jan F Meilof

**Affiliations:** 1 Nuclear Medicine and Molecular Imaging, University of Groningen, University Medical Center Groningen, Groningen, The Netherlands; 2 Radiology, University of Groningen, University Medical Center Groningen, Groningen, The Netherlands; 3 Department of Biomedical Sciences of Cells and Systems, University of Groningen, University Medical Center Groningen, Groningen, The Netherlands; 4 Faculty of Physics, University of Vienna, Vienna, Austria; 5 Computational Radiology Laboratory, Boston Children’s Hospital, Boston, USA; 6 Department of Radiology, Boston Children’s Hospital, Boston, MA, USA; 7 Harvard Medical School, Boston, MA, USA; 8 Intelligent Medical Imaging Research Group, Boston Children’s Hospital, Boston, MA, USA; 9 Department of Neurology, Martini Ziekenhuis, Groningen, The Netherlands

**Keywords:** Axonal integrity, diffusion kurtosis imaging, diffusion tensor imaging, fixel-based analysis, multiple sclerosis

## Abstract

Neuronal damage is the primary cause of long-term disability of multiple sclerosis (MS) patients. Assessment of axonal integrity from diffusion MRI parameters might enable better disease characterisation. 16 diffusion derived measurements from diffusion tensor imaging (DTI), diffusion kurtosis imaging (DKI), and fixel-based analysis (FBA) in lesions, peri-lesion and normal appearing white matter were investigated. Diffusion MRI scans of 11 MS patients were processed to generate DTI, DKI, and FBA images. Fractional anisotropy (FA) and fibre density (FD) were used to assess axonal integrity across brain regions. Subsequently, 359 lesions were identified, and lesion and peri-lesion segmentation was performed using structural T_1_w, T_2_w, T_2_w-FLAIR, and T_1_w post-contrast MRI. The segmentations were then used to extract 16 diffusion MRI parameters from lesion, peri-lesion, and contralateral normal appearing white matter (NAWM). The measurements for axonal integrity, DTI-FA, DKI-FA, FBA-FD, produced similar results. All diffusion MRI parameters were affected in lesions as compared to NAWM (p < 0.001), confirming loss of axonal integrity in lesions. In peri-lesions, most parameters, except FBA-FD, were also significantly different from NAWM, although the effect size was smaller than in lesions. The reduction in axonal integrity in peri-lesions, despite unaffected fibre density estimates, suggests an effect of Wallerian degeneration.

## Introduction

Multiple sclerosis (MS) is the most common neurodegenerative disease among young adults. In MS patients, the myelin sheath surrounding neuronal axons is attacked by the immune system, leading to demyelination.^1–3^^ ^As myelin has a neuroprotective and metabolic support function, axons become susceptible to deterioration upon demyelination, which can eventually lead to neurodegeneration.^4,5^ MS is a heterogeneous disease and multiple factors may contribute to the clinical symptoms in individual patients, including the number, size and location of demyelinated lesions and the severity of the axonal injury induced in these lesions. Axonal integrity is directly associated with neuronal function and measuring axonal integrity could therefore help to understand the patient’s symptoms and support disease prognosis.

Axonal integrity can be assessed with magnetic resonance imaging (MRI) by measuring the diffusion of water molecules. When physical boundaries are not in close proximity (like in the cerebral spinal fluid, CSF), the water molecules can diffuse freely in any direction which is called isotropic diffusion.^6^ However, when physical boundaries are present (like in white matter), the diffusion is hindered, which gives directionality to the diffusion of water molecules and therefore is called anisotropic diffusion. The proportion of anisotropic diffusion is called fractional anisotropy (FA). As axons give directionality to water diffusion, there is high FA in the white matter. However, FA varies along the axonal trajectory in white matter, since regions with many axonal fibre bundles usually have higher FA values, and regions with a low density of axons usually have lower FA values. This simplified explanation of FA, however, only applies when all axons run in the same direction. When axons are not aligned, but crossing in different directions, a lower FA will be observed and FA will therefore no longer represent axonal density.

Several models can be applied for modelling of diffusion MRI, under which diffusion tensor imaging (DTI), diffusion kurtosis imaging (DKI), and fixel based analysis (FBA). Among these, DTI is the most commonly applied method, but assumes linearity of the diffusion signal decay between b-values. At higher b-values, however, this concept of linear diffusion signal decay is no longer valid, as diffusion is restricted due to tissue. This deviation of linearity is called kurtosis, which can be estimated with DKI. With FBA, parameters like fibre density (FD), fibre bundle cross-section (FC), and fibre density and bundle cross section (FDC) can be estimated. A more thorough explanation of the parameter estimates with DTI, DKI, or FBA can be found in the methods section.

DTI is already well investigated in MS, showing decreased FA values in both active and inactive lesions as compared to tissue devoid of lesions, the so-called normal appearing white matter (NAWM).^7–9^ DKI has been used clinically for various pathologies, like ischemic stroke, Alzheimer’s disease, and oncology.^10–12^ However, its relevance for MS has not been fully elucidated yet,^11–13^ as literature regarding DKI in MS is scarce. Studies that used DKI in MS mainly investigated the trajectories of axonal bundles,^14–19^ but a thorough evaluation of the axonal integrity in white matter lesions (WML) using DKI-FA has not yet been performed. So far, only one study on FBA in MS patients assessed FD in WML, and showed a lower FD in WML as compared to NAWM, but did not investigate FC and FDC in WML.^20^

Since a comparison of all these diffusion parameters in the same patient has not been reported yet, this study aims to assess the similarities and discrepancies between four tensor (DTI-AD, DTI-RD, DTI-MD, and DTI-FA), nine kurtosis (DKI-AD, DKI-RD, DKI-MD, DKI-FA, AK, RK, MK, KFA, and MKT) and three fixel-based (FBA-FD, FBA-FC, FBA-FDC) parameters for estimating axonal integrity and the potential value of diffusion MRI derived parameters for characterization of not only WML, but also peri-lesion white matter in MS patients. Peri-lesion axonal integrity could provide information about the extent of the axonal damage and thus might be a prognostic marker. Therefore we compare DTI, DKI, and FBA derived parameters to explore the relevance of various diffusion MRI parameters for a better disease characterization in MS regarding both lesions and peri-lesions.

## Methods

### Subjects

Eleven MS patients, diagnosed according to the revised McDonald Criteria were included in this prospective study. The inclusion criteria were: at least 18 years old and a diagnosis of progressive MS. Exclusion criteria were: Pregnancy, breastfeeding, a previous adverse reaction to gadolinium injections, claustrophobia, cerebrovascular disease, a clinical history of diminished renal or liver function, current use of investigational medication in the context of a clinical trial, and common contra-indications for MRI-examination, such as the presence of magnetisable materials in the body. Written informed consent was obtained from all study participants. The study was approved by the medical ethics committee of the Medical University Center Groningen (METc-number: 2018/450) in accordance to the Helsinki Declaration.

### Image acquisition

All MRI scans were acquired on the same 3.0 Tesla scanner (Siemens Magnetom Prisma) equipped with a 64-channel head coil. The brain imaging protocol included the following sequences: a sagittal 3D T_1_w MP-RAGE (TR: 2300 ms; TE: 2.31 ms; TI: 900 ms; flip angle: 8°; slice thickness: 0.9 mm; voxel size: 0.9 × 0.9 × 0.9 mm, TA: 6:35), a sagittal 3D T_2_w-FLAIR SPACE (TR: 5000 ms; TE: 392 ms; TI: 1800 ms; flip angle: 90°, slice thickness: 0.9 mm; voxel size: 0.9 × 0.9 × 0.9 mm, TA: 6:22), a sagittal 3D T_2_w SPACE (TR: 3200 ms; TE: 408 ms; flip angle: 90°; slice thickness: 0.9 mm; voxel size: 0.4 × 0.4 × 0.9 mm, TA: 5:09), a DWI EPI (TR: 2200 ms; TE: 77 ms; flip angle: 90°; 60 transversal slices; AP phase encoding direction; 64 diffusion directions, b-values 0, 1000, and 2500 s/mm^2^; slice thickness: 2.5 mm; voxel size: 2.5 × 2.5 × 2.5 mm, partial Fourier 6/8, SMS acceleration factor slice 4, TA: 5:07), a DWI (TR: 2200 ms; TE: 77 ms; flip angle: 90°; 60 transversal slices; PA phase encoding direction;  × 64 diffusion directions, b-values 0 s/mm^2^; slice thickness: 2.5 mm; voxel size: 2.5 ×2.5 × 2.5 mm, partial Fourier 6/8, SMS acceleration factor slice 4, TA: 0:51), and a post-contrast (gadolinium 0.2 ml/kg) sagittal 3D T_1_w MPRAGE, with parameters identical to those of pre-contrast 3D T_1_w.

### Parameters of interest

The overall movement of a water molecule can be separated into three orthogonal directions of diffusion and thus expressed as a combination of the eigenvalues λ1, λ2, and λ3. λ1 is also known as axial diffusivity (AD), the diffusion in longitudinal direction, whereas radial diffusivity (RD) is the mean of λ2 and λ3, depicting sideward diffusion perpendicular to the longitudinal direction, and mean diffusivity (MD) is the mean of all three eigenvalues.

With diffusion tensor imaging (DTI), it is assumed that the water molecules do not interact with the physical boundaries, and therefore the diffusion of the water molecules is considered to be free, but hindered. These assumptions are valid when low b-values are applied. At low b-values, the signal is dominated by hindered diffusion (i.e. extra-axonal space), and therefore DTI is generally measured with a b-value of 1000 s/mm^2^. However, the assumptions of hindered diffusion do not hold for b-values larger than 1000 s/mm^2^. If the interaction of the diffusing water molecule to its surroundings is strong, e.g. when the water molecule is located in a very compact space which is smaller than the typical diffusion length, the diffusion process itself will be influenced and is hence called restricted.^6,21–23^ Diffusion kurtosis imaging (DKI) incorporates the information gathered by diffusion acquisitions with b-values of approx. 2000 s/mm^2^, and therefore also takes diffusion restriction into account in its estimates.^21^ We henceforth distinguish between DTI-AD, DTI-RD, DTI-MD, and DTI-FA, being AD, RD, MD, and FA derived from DTI analysis, and DKI-AD, DKI-RD, DKI-MD, and DKI-FA, as calculated with DKI analysis. In addition, when higher b-values (b > 2000 s/mm^2^) are used, the effect that kurtosis has on diffusivity parameters can be mapped, resulting in parameters called axial kurtosis (AK), radial kurtosis (RK), mean kurtosis (MK), kurtosis FA (KFA), and a parameter called mean kurtosis tensor (MKT).^10,21,24–26^ While AD is influenced by intra-cellular structures, mainly axons in case of the brain, AK is also thought to be influenced by intracellular structures. Similarly, whereas RD seems to be primarily affected by myelin, RK is also affected by structures perpendicular to the main diffusion direction, like myelin and other cellular membranes. However, direct biological interpretations of these parameters across pathologies in absence of a direct post-mortem comparison should be cautiously performed as different biological processes might have a similar diffusivity profile.^27^ In stroke, a sharp increase in AK was associated with tissue swelling, while RK was shown to be increased in Alzheimer’s disease.^10,11,13^ MK increases if the partition of water compartments, which are small enough to restrict water diffusion, increases. Therefore, higher grade tumours have a higher MK, presumably as a result of increased cellular density, decreased cell size, and increased complexity.^12^

Heuristic descriptors of the diffusion signal in MRI, such as FA, can be difficult to interpret in brain regions containing crossing fibre bundles within a voxel as the average FA in the voxel is not equal to the sum of the FA of the individual bundles anymore. With fixel-based analysis (FBA), the actual fibre density (FD) can be calculated^28^ which is supposed to be an even more accurate estimate for axonal integrity than FA as it is not sensitive to fibre orientated dispersion and crossings. In addition, the fibre bundle cross-section (FC) and the combination of fibre density and bundle cross-section (FDC) can be determined. Both a reduced number of fibres within a bundle, i.e. FD, and a reduced fibre bundle diameter, i.e. FC, can result in a decreased axonal function.

### Image analysis

The image analysis included the following steps: diffusion analysis for generation of parametric diffusion maps and co-registration to structural T_1_w; delineation of regions-of-interest (ROI) for extraction of diffusion parameters from tissues of interest; lesion classification for determination of lesion activity; tissue segmentation for generation of binary masks of WML, contra-lateral NAWM, and peri-lesion white matter. A schematic flow chart for the image processing procedure is shown in Appendix Fig. 1.

First noise reduction was applied to diffusion-weighted MRI.^29,30^ Images were corrected for Gibbs ringing artefacts,^31^ followed by field-map correction of susceptibility distortions,^32,33^ motion correction with correction for eddy-current induced distortions,^34–36^ and bias field correction.^37^ These corrected diffusion data were subsequently used to estimate the tensor parameters (DTI-AD, DTI-RD, DTI-MD, and DTI-FA) using b0 and b1000 volumes, the kurtosis parameters (DKI-AD, DKI-RD, DKI-MD, DKI-FA, AK, RK, MK, KFA, and MKT) using b0, b1000, and b2500 volumes, and the fixel parameters (FBA-FD, FBA-FC, FBA-FDC) using b0, b1000, and b2500 volumes. More specifically: The tensor parameters were calculated with FMRIB Software Library (FSL v6.0.4),^33^ the kurtosis parameters with diffusion kurtosis estimator (DKE v2.6.0),^26^ and the fixel parameters with MRtrix v3.0.^38^ In addition to the above described pre-processing steps, the generation of the fixel parameters required additional pre-processing. Multi-tissue constrained spherical deconvolution (CSD) was applied using tissue-specific response functions for white matter, grey matter, and cerebrospinal fluid,^39,40^ which was followed by up-sampling of the DWI data with a tricubic interpolation to an isotropic resolution of 1.25 mm. Then, the population-average fibre orientation distribution (FOD) was estimated using population averaged tissue-specific response functions,^41^ followed by intensity normalisation,^42^ and the generation of a population-averaged FOD template. The FOD images of each subject were then registered to the FOD template, to create a template mask. The masked FOD images of each subject were warped to the template space, and segmented^28^ to estimate fixels and the apparent fibre density (FD).^43^ The fixels were then reoriented, after which the fixels of a subject were assigned to template fixels, and finally the FC and FDC were calculated.^44^ The FD, FC, and FDC fixel images were then converted to voxel images and transformed from template to subject space, in order to be able to compare all diffusion derived parameters within the same space. The images resulting from DTI, DKI, and FBA analysis were subsequently co-registered to T_1_w MRI using SPM12 (Wellcome Trust Centre for Neuroimaging, Institute of Neurology, London, 2014).^45^ Brain parcellation using the regions of the Hammers atlas on DTI-FA, DKI-FA, and FBA-FD parametric images was performed in PMOD v4.1 (PMOD technologies, Zurich, Switzerland) to obtain ROI estimates of axonal integrity.

Tissue segmentation was performed to derive binary masks representing WML, peri-lesion white matter, contra-lateral NAWM, white matter (WM), and grey matter (GM). WML segmentation was performed using T_1_w, T_2_w, and T_2_w-FLAIR MRI as previously described.^46^ In brief, T_1_w, T_2_w, and T_2_w-FLAIR were used for lesion segmentation based on a convolutional deep neural network with an asymmetric similarity loss function. The asymmetric similarity loss function mitigates the imbalance between the number of non-lesion voxels and lesion voxels, thereby achieving a better trade-off between precision and recall than other algorithms. This resulted in WML probability maps, which were manually adjusted to correspond with WML, if necessary. Contralateral NAWM regions, devoid of any structural damage as assessed with T_1_w and T_2_w-FLAIR, were generated using PMOD. Peri-lesion white matter regions were generated in PMOD by increasing the size of the region of a WML by 3 voxels (corresponding with ∼3 mm) in all directions and subsequently subtracting the WML. ROI analysis was applied to extract diffusion parameters from the corresponding WML, peri-lesion white matter, and NAWM. In addition, T_1_w MRI was segmented with SPM12 to obtain GM and WM masks with a probability threshold of 0.9 to mitigate a bias in the GM and WM segmentations caused by WML. These GM and WM masks were added to the VOIs of the Hammer’s atlas to obtain regional values for the DTI-FA, DKI-FA, and FBA-FD parametric images. First, only the GM and WM were used to determine whether the various parametric images could discriminate between regions with different densities in axons. This was followed by correlational analysis using the 144 brain regions of the Hammer’s atlas to determine whether the diffusion MRI methods (DTI-FA, DKI-FA, and FBA-FD) for assessment of axonal integrity produced related results.

### Lesion classification

The lesions were radiologically classified using T_1_w, T_2_w, T_2_w-FLAIR, and contrast-enhanced T_1_w MRI. WML were detected as hyperintense lesions on T_2_w and T_2_w-FLAIR images. Their activity status was determined using contrast-enhanced T_1_w images. When lesions were hyperintense on T_1_w due to contrast-enhancement, they were considered as active lesions. When lesions were not hyperintense on contrast-enhanced T_1_w images, the lesions were classified as inactive lesions.^1^

### Statistical analysis

To assess whether DTI, DKI, and FBA indeed give different estimates of axonal integrity in regions with known differences in axonal integrity, the results from DTI-FA, DKI-FA, and FBA-FD for GM and WM were compared with each other. In addition, correlational analysis was performed to determine whether DTI-FA, DKI-FA, and FBA-FD indeed differ from each other in assessing axonal integrity within various regions across the whole brain. For correlation analysis, Spearman correlation was used, because the data was not normally distributed.

To evaluate the potential value of all 16 diffusion measures for lesion characterization, the diffusion measures for WML and peri-lesions were compared to NAWM.

For the aforementioned analyses, either non-parametric (Mann-Whitney or Kruskal Wallis) or parametric tests (students’ t-test or ANOVA) were applied, depending on normality of the data, as assessed with the Kolmogorov-Smirnov test. Significance level was set to p < 0.05 for all analyses. Statistical analysis was performed in SPSS Statistics, version 23 (IBM, Chicago IL, USA). As this study is of exploratory nature for assessing the value of diffusion MRI parameters for MS prognosis and disease understanding, no corrections for multiple comparisons were performed as subtle effects might then be missed.

## Results

### Demographics

Eleven MS patients were recruited in this study, of which 6 had secondary progressive MS (SPMS) and 5 had primary progressive MS (PPMS). The demographics of the patients are provided in Appendix Table 1. The mean age was 52.5 (SD ± 8.0) years, and 73% of the subjects were male. Patients had at least 5 years the corresponding diagnosis and did not receive disease modifying treatments.

### DTI-FA, DKI-FA, and FBA-FD in brain regions

**Figure 1.**
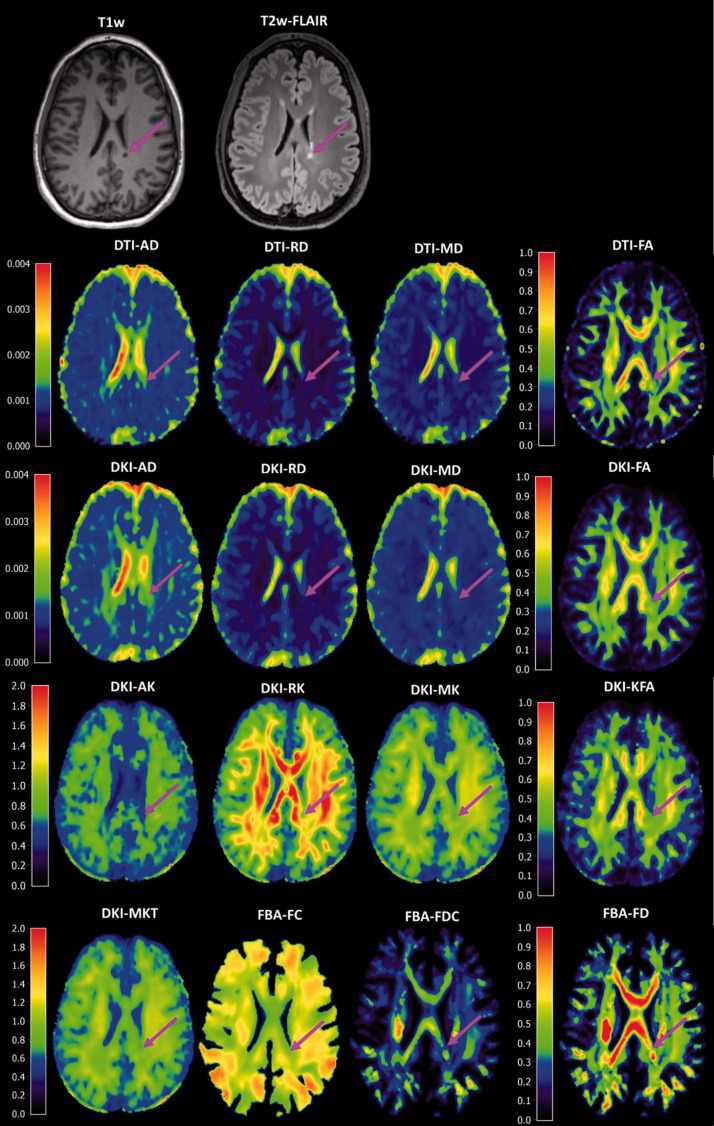
Structural T_1_w and T_2_w MRI with various diffusion MRI derived parameters. The brain figures are from one subject, with a black hole (pink arrow) on the presented slice. Close-up images of the lesion sites are provided in Appendix Figure 3.

For assessing WML and peri-lesion WM with parameters of axonal integrity, one should first assess whether these axonal parameters are indeed corresponding with biological expectations. As histopathology is not possible with *in vivo* human imaging, one should assess whether the parameters are corresponding with known variances in axonal density across regions. The GM is generally considered to be dominated with neuronal cell bodies and the WM with axons. The Kruskal-Wallis tests showed that the DTI-FA, DKI-FA, and FBA-FD estimates (Figure 1) were significantly different between GM and WM (p < 0.001, Table 1). The U value of all three parameters for distinguishing WM and GM was the same, indicating that the differentiation between these regions was comparable for these diffusion parameters.

**Table 1.** Comparison of grey matter (GM) and white matter (WM) with various measures for axonal integrity.

	Grey matter	White matter	U	p-value
DTI-FA	0.141 (±0.0023)	0.379 (±0.017)	121	<0.001
DKI-FA	0.148 (±0.0038)	0.376 (±0.018)	121	<0.001
FBA-FD	0.109 (±0.018)	0.3937 (±0.025)	121	<0.001

Data are shown as average (± standard deviation).

**Figure 2.**
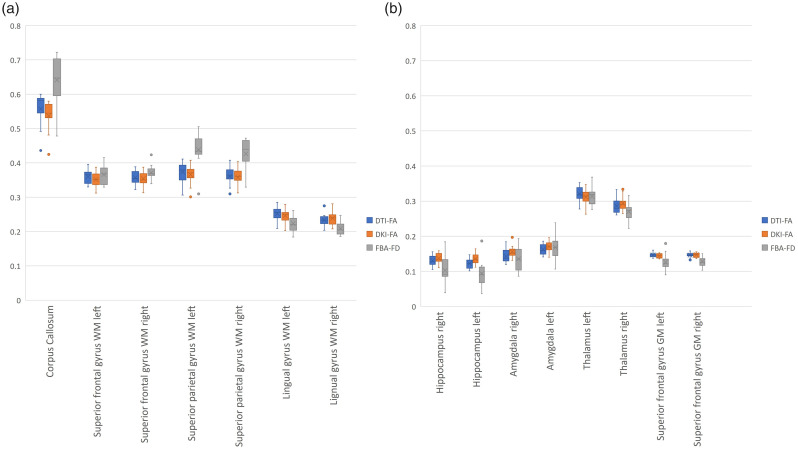
Distribution plots of DTI-FA, DKI-FA, and FBA-FD per brain region. Representative brain regions are chosen for temporal lobe (hippocampus and amygdala), frontal lobe (superior frontal gyrus), occipital lobe (lingual gyrus), parietal lobe (superior parietal gyrus), and central structures (corpus callosum and thalamus) for both WM (a), GM (b), left, and right, if applicable.

**Figure 3.**
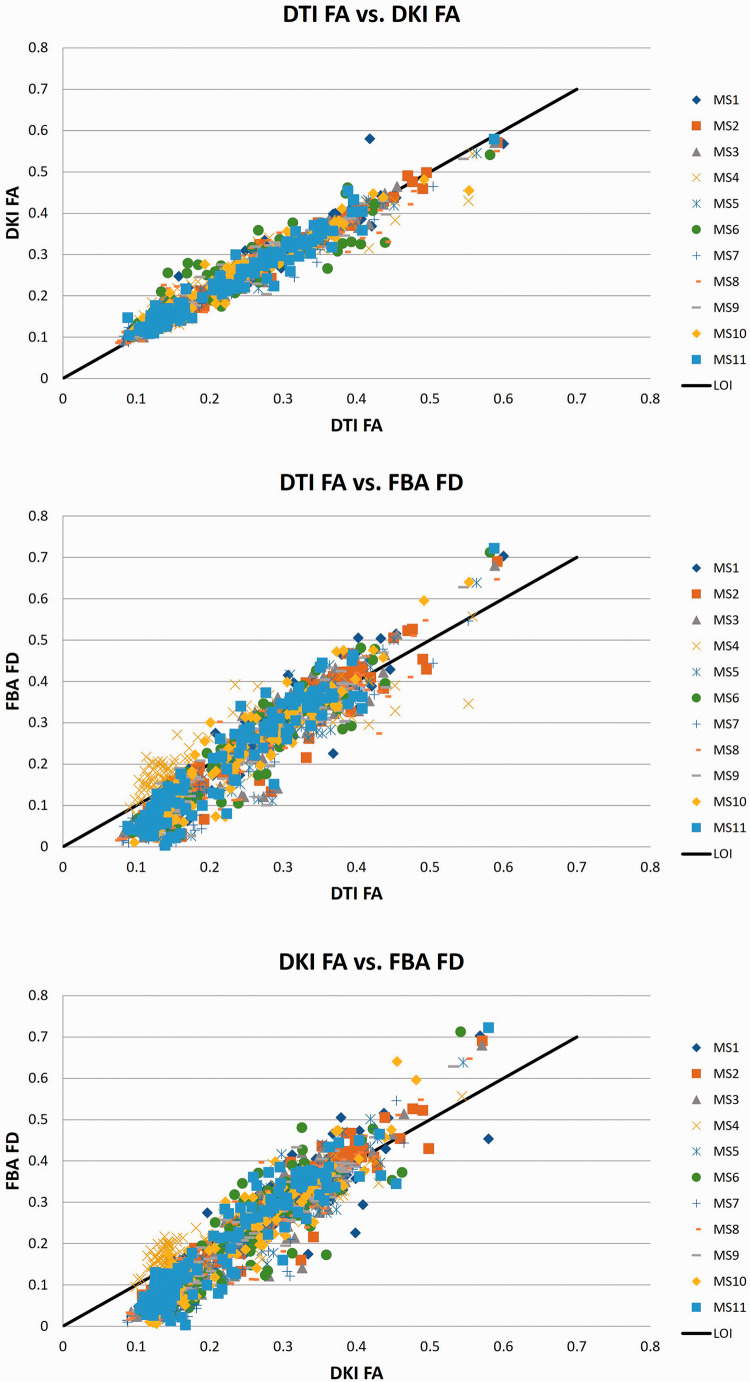
Correlations between axonal integrity parameters of DTI, DKI, and FBA.

Spearman correlations over all 144 brain regions of the Hammers atlas showed strong relationships between DTI-FA, DKI-FA, and FBA-FD (Figures 2 and 3 and Appendix Table 2). A correlation of 0.97 (p < 0.001) between DTI-FA and DKI-FA, a correlation of 0.91 (p < 0.001) between DTI-FA and FBA-FD, and a correlation of 0.90 (p < 0.001) between DKI-FA and FBA-FD were observed.

### MS lesion and peri-lesion characterization

A total of 359 lesions were identified, covering a total volume of 62.9 ml (Appendix Table 1). Of the 359 lesions, 3 were radiologically classified as non-MS lesion and therefore not used in subsequent analysis. None of these lesions were hyperintense on T_1_w post gadolinium MRI scans, and therefore all lesions were classified as inactive lesions. The patient with the lowest number of lesions had 8 lesions, and the patient with the highest number of lesions had 78 lesions with a median lesion number of 22 (IQR = 14–48.5, Appendix Fig. 2). The lesion numbers of the other patients were evenly distributed between the lowest and highest number of lesions. First correlations between all 16 parameters for both SPMS and PPMS were performed (Appendix Tables 3–4), and subsequently differences between SPMS and PPMS were assessed for both black holes and other MS lesions for all 16 parameters (Appendix Table 5). For black holes, DTI-AD, DTI-RD, DTI-MD, DKI-AD, DKI-RD, and DKI-MD were significantly lower in SPMS than in PPMS, whereas KFA was significantly higher. For other WML, DTI-AD, DTI-MD, DTI-FA, DKI-AD, DKI-FA, RK, FD, and FDC were significantly lower in SPMS than in PPMS, whereas AK was significantly lower. When assessing in SPMS differences between black holes, other WML, peri-lesion, and contra-lateral NAWM, all parameters were significantly different except for FC (Appendix Tables 6–7). In PPMS, all parameters were significantly different between black holes, other WML, peri-lesion, and contra-lateral NAWM (Appendix Tables 8-9).

**Figure 4.**
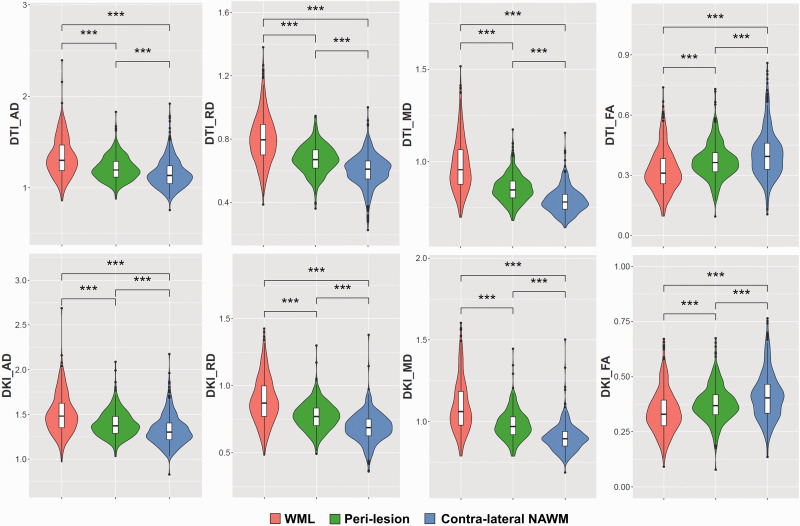
Heuristic diffusion MRI parameters.

**Figure 5.**
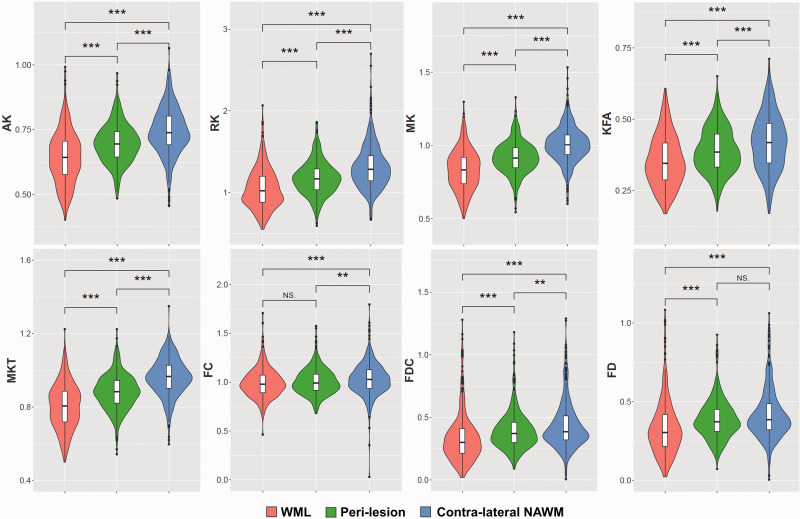
Kurtosis and fixel-based diffusion MRI parameters.

The ability to discriminate WML from NAWM was assessed for all 16 parameters derived from the tensor, kurtosis, and fixel-based analysis methods (Figures 4 and 5, Table 2, appendix Table 10). Among the parameters derived from tensor analysis (Figure 1), the diffusivity measures DTI-AD, DTI-RD, and DTI-MD were significantly higher in lesions than in NAWM (p < 0.001), whereas the anisotropy measure DTI-FA was significantly lower in lesions than in NAWM (p < 0.001). Among the kurtosis parameters, the diffusivity measurements (DKI-AD, DKI-RD, DKI-MD) were significantly higher in WML than in NAWM (p < 0.001), while DKI-FA, and the kurtosis effects (AK, RK, MK, KFA, and MKT) were significantly lower (p < 0.001). All fibre parameters derived from the FBA (FC, FD, and FDC) were significantly lower in lesions compared to NAWM (p < 0.001). In short, the parameters suggest a significantly higher diffusivity and a significantly lower fractional anisotropy and fibre density in lesions than in NAWM.

**Table 2.** Lesion and peri-lesion diffusion parameters.

Diffusion parameter	Lesion	Peri-lesion	NAWM
Tensor^a^			
DTI-AD	1.33 (±0.21)	1.21 (±0.13)	1.16 (±0.16)
DTI-RD	0.80 (±0.16)	0.67 (±0.09)	0.60 (±0.10)
DTI-MD	0.98 (±0.15)	0.85 (±0.07)	0.79 (±0.07)
DTI-FA	0.33 (±0.11)	0.38 (±0.10)	0.41 (±0.12)
Kurtosis^a^			
DKI-AD	1.51 (±0.22)	1.39 (±0.14)	1.33 (±0.17)
DKI-RD	0.89 (±0.17)	0.78 (±0.10)	0.69 (±0.11)
DKI-MD	1.09 (±0.16)	0.98 (±0.09)	0.90 (±0.08)
DKI-FA	0.34 (±0.10)	0.38 (±0.081)	0.41 (±0.11)
AK	0.64 (±0.10)	0.79 (±0.08)	0.74 (±0.09)
RK	1.05 (±0.24)	1.17 (±0.21)	1.32 (±0.27)
MK	0.83 (±0.14)	0.92 (±0.11)	1.01 (±0.12)
KFA	0.35 (±0.09)	0.39 (±0.08)	0.42 (±0.10)
MKT	0.80 (±0.12)	0.88 (±0.10)	0.96 (±0.10)
Fixel^a^			
FC	0.99 (±0.15)	1.01 (±0.13)	1.03 (±0.17)
FD	0.33 (±0.18)	0.39 (±0.12)	0.42 (±0.16)
FDC	0.34 (±0.20)	0.39 (±0.15)	0.44 (±0.19)

Data are shown as average (±standard deviation).

^a^All parameters were significantly different (p < 0.05) between lesion, peri-lesion, and NAWM, except for the difference in FBA-FD between peri-lesion and NAWM and the difference in FBA-FC between lesion and peri-lesion (see Appendix Table 10 and 11).

The trend in diffusion MRI parameters that was observed for lesions was also found for peri-lesions (Figures 4 and 5, Table 2, appendix Table 11), although the effect in peri-lesions was smaller than in lesions. Yet, the differences between peri-lesion and NAWM were statistically significant for all diffusion parameters, except for FBA-FD. When comparing the various parameters between lesion and peri-lesion (appendix Table 12), all differences were significant as well, except for FBA-FC (p = 0.17).

## Discussion

This study aimed to investigate the potential value of diffusion MRI derived parameters for WML and peri-lesion characterization. A better WML and peri-lesion characterization could facilitate the understanding of clinical symptoms and improve disease prognosis of patients with MS. Our findings show that the various diffusion MRI derived proxies for axonal integrity (DTI-FA, DKI-FA, and FBA-FD) correlate strongly with each other. The FBA parameters provided interesting supplementary information on structural changes, as compared to the commonly used diffusion parameters AD, RD, MD, and FA.

When comparing the different strategies for assessing axonal integrity across the different diffusion MRI analysis methods, DTI-FA, DKI-FA, and FBA-FD performed comparably. All three methods were significantly different between GM and WM, known regions with differential axonal densities. In addition, the correlational analysis using 144 brain regions per subject, showed strong correlations between the methods, indicating that all three methods for axonal integrity measure the same biological phenomenon, which was expected. These very high correlations suggest that the different strategies to optimize the estimation of axonal integrity with diffusion MRI had only minimal effect. On the other hand, the differences could also be too subtle to measure on an ROI level and that analysis of smaller regions, e.g. lesions, may be required to show any differences between the methods. However, also at lesion level (appendix Table 3-4) very strong correlations were observed among the parameters for axonal integrity.

At lesion level, our findings of increased diffusivity with the tensor based methods (DTI-AD, DTI-RD, DTI-MD) is in agreement with current literature showing increased diffusivity in MS lesions.^7–9,47^ This even led to the discussion about whether increased diffusivity could be a marker for lesion activity.^47^ However, in our study increased diffusivity is even observed in lesions that are classified as inactive according to the traditional method with post-contrast T_1_w images. The increase in diffusivity (AD, RD and MD) within lesions, as observed in both DTI and DKI analyses, is in agreement with a decrease in physical boundaries that occurs during degenerative processes in MS lesions. This corresponds with the findings that FA in lesions is decreased, which indicates a reduced directionality in diffusion and thus an increase in isotropic diffusion. A decrease of FA and increase of diffusivity within lesions is also in agreement with previous studies using DTI to assess FA, AD, RD, and MD in WML^7–9^, however, differences between active and inactive WML were less clear.^9^ Taken together, these results indicate that the potential use of diffusion MRI for determining lesion activity requires further investigation, since increased diffusivity was also observed in inactive lesions. An increase in diffusivity in active lesions could be caused by an increased amount of fluid and inflammatory cells in the lesion. If so, active lesions would present higher levels of diffusivity due to the combination of loss of physical boundaries and increased inflammatory activity, whereas inactive lesions would show the lower levels of increased diffusivity due to only the loss of physical boundaries.

The findings of decreased DTI-FA and DKI-FA within lesions and peri-lesions, suggests that there are no real differences between DTI-FA and DKI-FA for assessing axonal integrity. This is in contrast to the hypothesis that the integration of higher b-values (b > 2000 s/mm^2^) within the DKI estimates would be more biologically accurate than tensor based methods. Nonetheless, DKI has been shown to be more sensitive in extreme cases of brain damage.^48–51^ The absence of differences between the methods might be due to alterations in micro structure being so extensive that changes can be observed with any diffusion MRI parameter. In our study all DTI and DKI diffusion MRI parameters that we investigated seem to be able to show structural alterations not only in lesions, but also in peri-lesion. However, all parameters seem to be less affected in peri-lesion than lesion. This suggests that the observed damage in peri-lesion is not as severe as in lesion.

The FBA showed a reduction in FC, FD, and FDC in MS lesions, which illustrates that fibre bundles are both severed in diameter and density, in agreement with the MS pathogenesis cascade. A phenomenon related to axonal damage in MS is Wallerian degeneration.^52–55^ Wallerian degeneration describes a phenomenon, in which focal axonal damage leads to distal axonal degeneration. Within peri-lesions the number of fibres in a bundle (FD) was not significantly affected, but the fibre diameter (FC and FDC) was reduced, which might be due to the Wallerian degeneration effect that could occur when axons within MS lesions are damaged, leading to degeneration of the axons in the peri-lesions.

With diffusion MRI, several indices for axonal integrity can be generated. We did not find any published literature that assessed all three diffusion MRI analysis methods within a single dataset for either axonal integrity or WML characterization. Moreover, none of these methods have been compared with histological findings for axonal integrity in WML in MS, and neither have other diffusion MRI parameters. However, a study investigating FA and axonal density with histopathology found that the FA was decreased in WML as compared to NAWM and axonal density was increased in WML as compared to NAWM.^56^ While these findings were not correlated with each other and therefore no firm statements can be made, the findings illustrate the difficulties with the interpretation of FA. Therefore, diffusion MRI derived indices require further validation, in particular a head-to-head comparison with independent pathological measures.

A limitation of our study is the absence of active lesions, as determined with gadolinium enhancement. This is most likely due to enrolment of solely progressive MS patients. Inclusion of relapse-remitting MS (RRMS) would most probably include patients with some active lesions as well, as the prevalence of active lesions is higher in RRMS than in progressive MS.^57^ Because of the lack of active lesions in our analysis we could not determine the discriminative power of diffusion derived parameters between active and inactive lesions. If active lesions were included, the sensitivity of the diffusion MRI derived parameters for microstructural changes could be further investigated, since active lesions have different biological constituents than inactive lesions.^58^^ ^As RRMS patients recover at least partially from clinical symptoms, one might expect that neuronal damage is less severe in RRMS than in PMS and therefore markers of neuronal integrity should be less affected in RRMS than in PMS. However, the inflammatory processes in RRMS are more prevalent than in PMS, and likely have major impact on the accuracy of diffusion parameter estimations. Therefore, the parameter estimations in PMS might be more reliable and robust. Neuronal degeneration is thought to be the major cause of clinical complaints in progressive MS and consequently the investigation of axonal integrity parameters appears more relevant for disease prognosis in progressive MS. The number of included subjects (N = 11) could be considered a limitation. Nevertheless with a total of 359 lesions, the impact is considered minimal.

In conclusion, all diffusion MRI parameters for axonal integrity are significantly different between regions with known differences in axonal integrity. Moreover, the diffusion parameters are strongly correlated with each other. For WML characterization the individual diffusion MRI parameters can give supplementary information with regard to the underlying biological processes. The diffusion MRI parameters indicate that alterations in lesion microstructure extend to peri-lesion areas, illustrating that MS induced damage extends beyond the surface of WML. The observation that fibre density is preserved in peri-lesions, but that the fibre cross section is decreased, might indicate Wallerian degeneration. These results illustrate that tensor, or kurtosis analysis should be preferably combined with fixel-based analyses for a thorough evaluation of the underlying pathogenesis in WML and peri-lesion white matter.

## Supplemental Material

sj-pdf-1-jcb-10.1177_0271678X221107953 - Supplemental material for Diffusion-derived parameters in lesions, peri-lesion, and normal-appearing white matter in multiple sclerosis using tensor, kurtosis, and fixel-based analysis Click here for additional data file. Supplemental material, sj-pdf-1-jcb-10.1177_0271678X221107953 for Diffusion-derived parameters in lesions, peri-lesion, and normal-appearing white matter in multiple sclerosis using tensor, kurtosis, and fixel-based analysis by Chris WJ van der Weijden, Anouk van der Hoorn, Jan Hendrik Potze, Remco J Renken, Ronald JH Borra, Rudi AJO Dierckx, Ingomar W Gutmann, Hakim Ouaalam, Davood Karimi, Ali Gholipour, Simon K Warfield, Erik FJ de Vries Jan F Meilof in Journal of Cerebral Blood Flow & Metabolism
