# Supplementary material for: Diffusion-derived parameters in lesions, peri-lesion and normal-appearing white matter in multiple sclerosis using tensor, kurtosis and fixel-based analysis
Source: J Cereb Blood Flow Metab. 2022 Jun 25;42(11):2095–106. doi: 10.1177/0271678X221107953 (PMC9580168; doi:10.1177/0271678X221107953)
Supplement: sj-pdf-1-jcb-10.1177_0271678X221107953 - Supplemental material for Diffusion-derived parameters in lesions, peri-lesion, and normal-appearing white matter in multiple sclerosis using tensor, kurtosis, and fixel-based analysis [file sj-pdf-1-jcb-10.1177_0271678X221107953.pdf]

Appendix

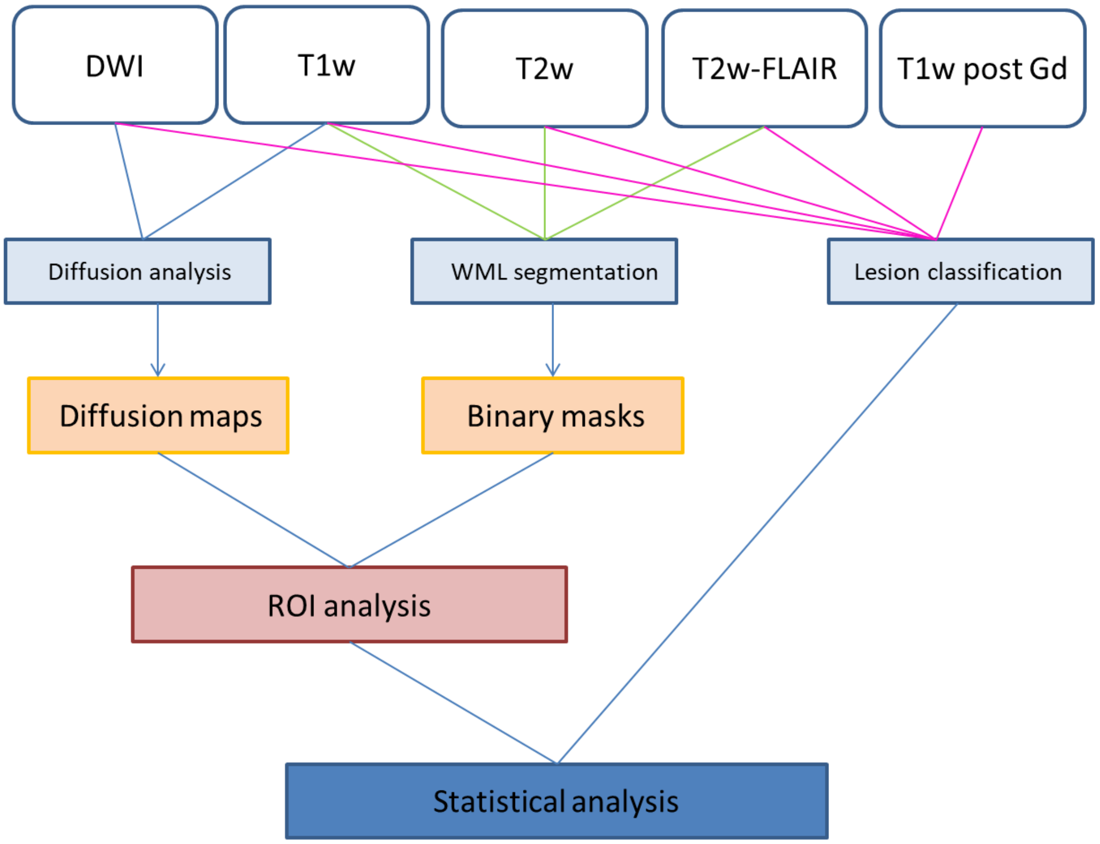

Figure 1: Schematic data-analysis flowchart

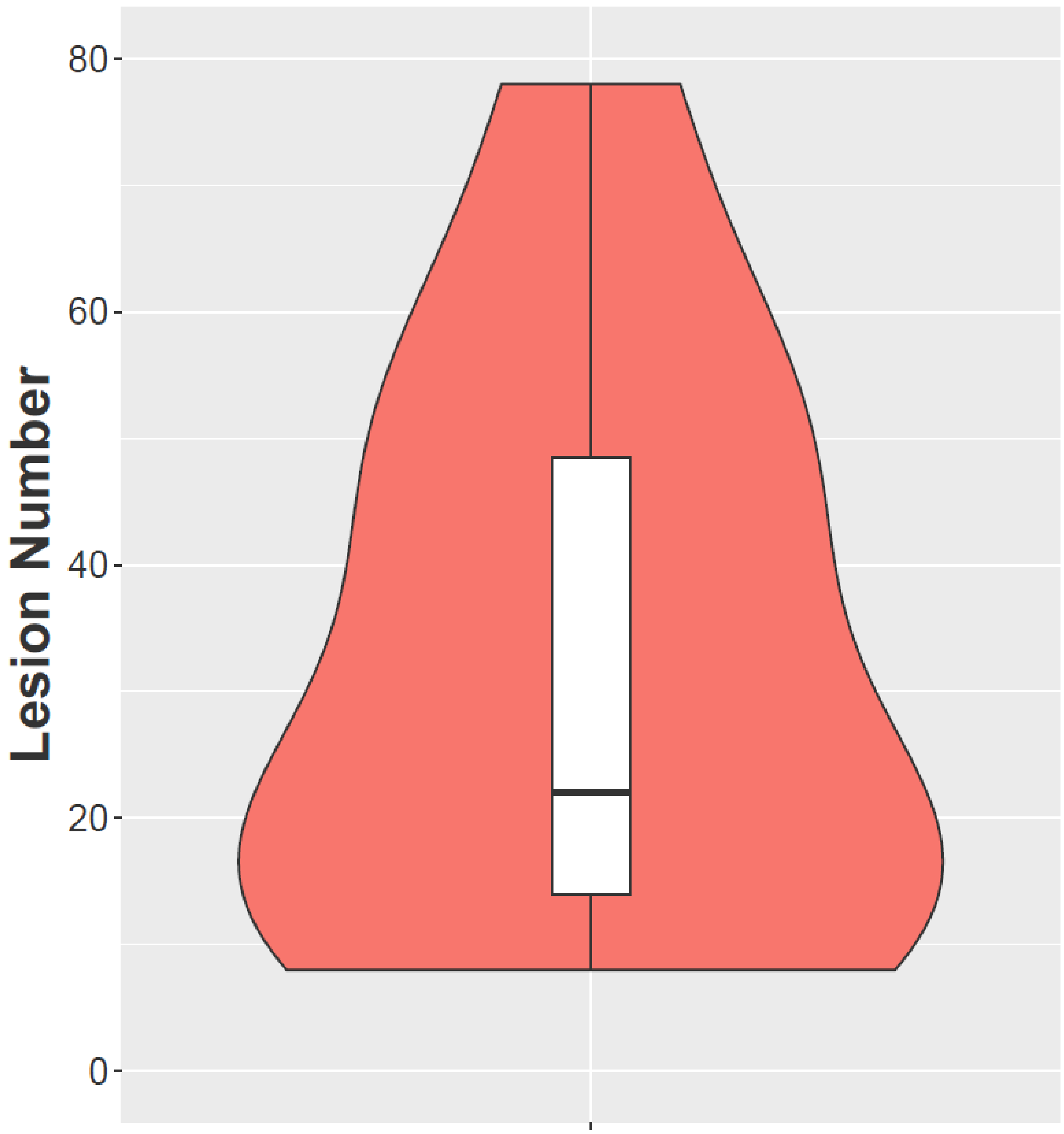

Figure 2: Lesion number distribution across subjects

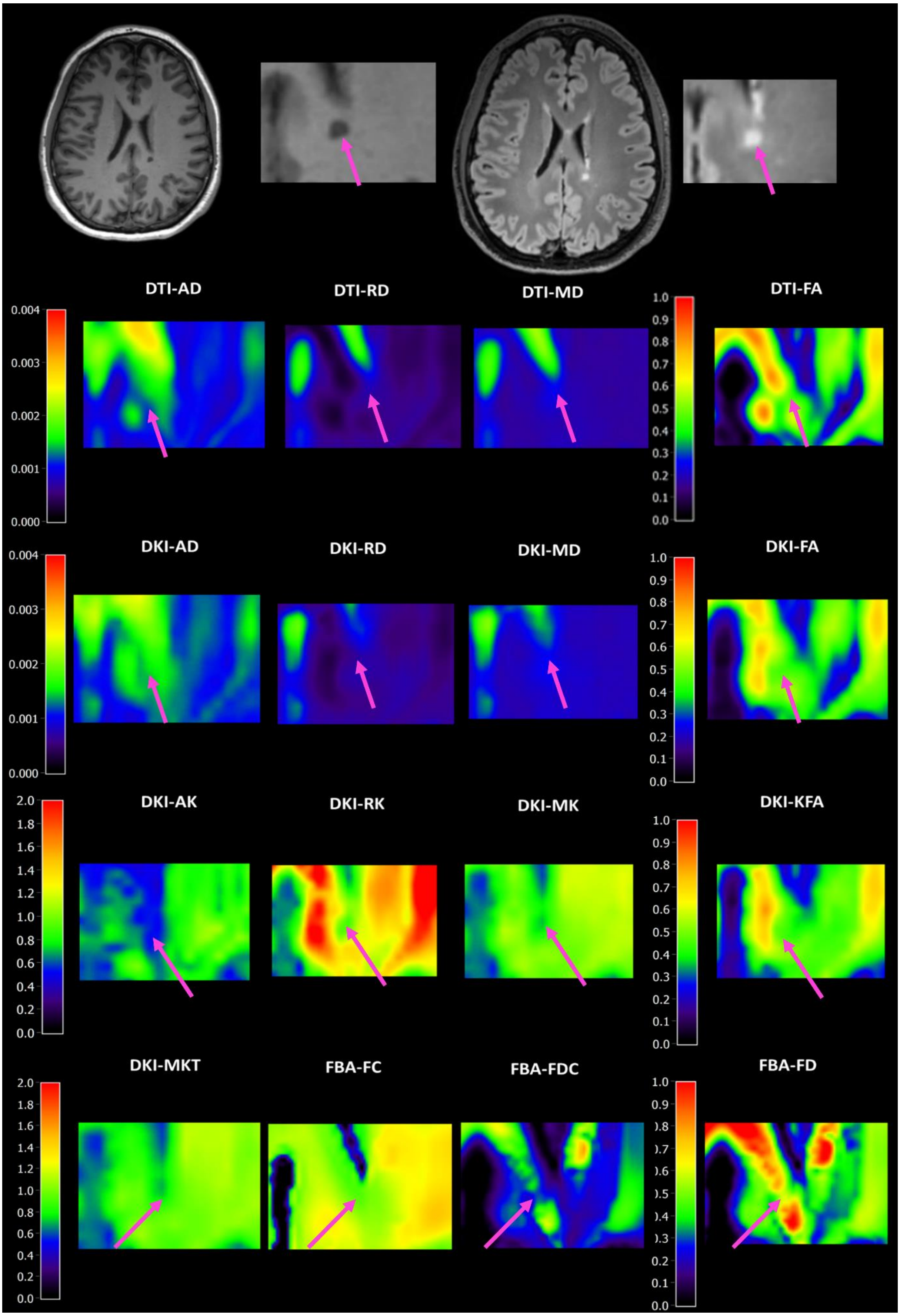

Figure 3: Close-up view of structural T1w and T2w MRI with various diffusion MRI derived parameters of a black hole (pink arrow).

Table 1: Demographics of the study participants.

Age (y)	52.5 (±8.0)
Male (%)	72.7%
PPMS (n)	5
SPMS (n)	6
Total number of lesions (n)	359
Average number of lesions (n)	33 (±23)
Average lesion volume (ml)	5.7 (±7.5)
Number of contrast enhanced lesions (n)	0

Data are shown as average (± standard deviation)

Table 2: Correlational analysis between DTI-FA, DKI-FA, FBA-FD across brain regions

Correlations

			DTI_FA	DKI_FA	FBA_FD
Spearman's rho	DTI_FA	Correlation Coefficient	1.000	0.965**	0.906**
		Sig. (2-tailed)	.	<0.001	<0.001
		N	1573	1573	1573
	DKI_FA	Correlation Coefficient	0.965**	1.000	0.899**
		Sig. (2-tailed)	<0.001	.	<0.001
		N	1573	1573	1573
	FBA_FD	Correlation Coefficient	0.906**	0.899**	1.000
		Sig. (2-tailed)	<0.001	<0.001	.
		N	1573	1573	1573

**. Correlation is significant at the 0.01 level (2-tailed).

Table 3: Significant Spearman correlation coefficients between diffusion MRI parameters of SPMS lesions

	DTI-AD	DTI-RD	DTI-MD	DTI-FA	DKI-AD	DKI-RD	DKI-MD	DKI-FA	AK	RK	MK	KFA	MKT	FD	FC	FDC
DTI-AD	-	0.35	0.68	0.33	0.90	0.34	0.64	0.20	-0.66	-0.24	-0.43	n.s.	-0.50	n.s.	0.20	0.16
DTI-RD	0.35	-	0.91	-0.68	0.33	0.93	0.84	-0.70	-0.42	-0.71	-0.70	-0.64	-0.66	-0.16	-0.63	-0.62
DTI-MD	0.68	0.91	-	-0.37	0.61	0.85	0.93	-0.44	-0.58	-0.64	-0.71	-0.47	-0.71	-0.15	-0.39	-0.40
DTI-FA	0.33	-0.68	-0.37	-	0.28	-0.64	-0.37	0.90	n.s.	0.53	0.40	0.71	0.33	n.s.	0.82	0.78
DKI-AD	0.90	0.33	0.61	0.28	-	0.36	0.68	0.24	-0.72	-0.25	-0.44	n.s.	-0.52	n.s.	0.19	0.14
DKI-RD	0.34	0.93	0.85	-0.64	0.36	-	0.91	-0.76	-0.42	-0.76	-0.73	-0.71	-0.69	-0.16	-0.63	-0.61
DKI-MD	0.64	0.84	0.93	-0.37	0.68	0.91	-	-0.47	-0.62	-0.68	-0.74	-0.51	-0.74	-0.17	-0.39	-0.41
DKI-FA	0.20	-0.70	-0.44	0.90	0.24	-0.76	-0.47	-	n.s.	0.62	0.48	0.82	0.40	n.s.	0.80	0.76
AK	-0.66	-0.42	-0.58	n.s.	-0.72	-0.42	-0.62	n.s.	-	0.57	0.75	0.21	0.81	n.s.	n.s.	n.s.
RK	-0.24	-0.71	-0.64	0.53	-0.25	-0.76	-0.68	0.62	0.57	-	0.86	0.60	0.83	0.08	0.50	0.49
MK	-0.43	-0.70	-0.71	0.40	-0.44	-0.73	-0.74	0.48	0.75	0.86	-	0.46	0.99	0.13	0.41	0.42
KFA	n.s.	-0.64	-0.47	0.71	n.s.	-0.71	-0.51	0.82	0.21	0.60	0.46	-	0.40	n.s.	0.56	0.53
MKT	-0.50	-0.66	-0.71	0.33	-0.52	-0.69	-0.74	0.40	0.81	0.83	0.99	0.40	-	n.s.	0.34	0.35
FD	n.s.	-0.16	-0.15	n.s.	n.s.	-0.16	-0.17	n.s.	n.s.	n.s.	0.13	n.s.	n.s.	-	0.19	0.40
FC	0.20	-0.63	-0.39	0.82	0.19	-0.63	-0.39	0.80	n.s.	0.50	0.41	0.56	0.34	0.19	-	0.96
FDC	0.16	-0.62	-0.40	0.78	0.14	-0.61	-0.41	0.76	n.s.	0.49	0.42	0.53	0.35	0.40	0.96	-

Table 4: Significant Spearman correlation coefficients between diffusion MRI parameters of PPMS lesions

	DTI-AD	DTI-RD	DTI-MD	DTI-FA	DKI-AD	DKI-RD	DKI-MD	DKI-FA	AK	RK	MK	KFA	MKT	FD	FC	FDC
DTI-AD	-	0.34	0.74	0.27	0.95	0.35	0.69	0.22	-0.75	-0.21	-0.45	n.s.	-0.54	n.s.	0.32	0.31
DTI-RD	0.34	-	0.85	-0.74	0.34	0.95	0.84	-0.76	-0.28	-0.74	-0.64	-0.72	-0.58	n.s.	-0.57	-0.54
DTI-MD	0.74	0.85	-	-0.36	0.72	0.82	0.94	-0.40	-0.58	-0.60	-0.67	-0.46	-0.68	n.s.	-0.21	-0.19
DTI-FA	0.27	-0.74	-0.36	-	0.23	-0.71	-0.39	0.96	n.s.	0.63	0.37	0.78	0.26	n.s.	0.86	0.81
DKI-AD	0.95	0.34	0.72	0.23	-	0.39	0.74	0.19	-0.76	-0.21	-0.45	n.s.	-0.54	n.s.	0.28	0.28
DKI-RD	0.35	0.95	0.82	-0.71	0.39	-	0.89	-0.77	-0.32	-0.75	-0.66	-0.74	-0.61	n.s.	-0.56	-0.53
DKI-MD	0.69	0.84	0.94	-0.39	0.74	0.89	-	-0.45	-0.57	-0.63	-0.68	-0.50	-0.68	n.s.	-0.26	-0.23
DKI-FA	0.22	-0.76	-0.40	0.96	0.19	-0.77	-0.45	-	n.s.	0.68	0.44	0.82	0.34	n.s.	0.85	0.80
AK	-0.75	-0.28	-0.58	n.s.	-0.76	-0.32	-0.57	n.s.	-	0.48	0.71	n.s.	0.80	n.s.	n.s.	n.s.
RK	-0.21	-0.74	-0.60	0.63	-0.21	-0.75	-0.63	0.68	0.48	-	0.93	0.42	0.86	-0.17	0.61	0.52
MK	-0.45	-0.64	-0.67	0.37	-0.45	-0.66	-0.68	0.44	0.71	0.93	-	0.23	0.98	-0.24	0.38	0.28
KFA	n.s.	-0.72	-0.46	0.78	n.s.	-0.74	-0.50	0.82	n.s.	0.42	0.23	-	n.s.	0.29	0.60	0.63
MKT	-0.54	-0.58	-0.68	0.26	-0.54	-0.61	-0.68	0.34	0.80	0.86	0.98	n.s.	-	-0.24	0.28	0.19
FD	n.s.	n.s.	n.s.	n.s.	n.s.	n.s.	n.s.	n.s.	n.s.	-0.17	-0.24	0.29	-0.24	-	n.s.	0.25
FC	0.32	-0.57	-0.21	0.86	0.28	-0.56	-0.26	0.85	n.s.	0.61	0.38	0.60	0.28	0.00	-	0.95
FDC	0.31	-0.54	-0.19	0.81	0.28	-0.53	-0.23	0.80	n.s.	0.52	0.28	0.63	0.19	0.25	0.95	-

Table 5: Comparison of black holes and WML between SPMS and PPMS

Diffusion parameter		Black holes				WML			
		SPMS (n=56)	PPMS (n=58)	U	Significance (p-value)	SPMS (n=165)	PPMS (n=77)	U	Significance (p-value)
Tensor									
	DTI-AD	1.29±0.17	1.44±0.22	997	**<0.001***	1.27±0.19	1.41±0.23	4060	**<0.001***
	DTI-RD	0.76±0.16	0.88±0.18	1033.5	**0.001***	0.79±0.13	0.80±0.17	6432	0.875
	DTI-MD	0.94±0.13	1.07±0.16	883	**<0.001***	0.95±0.13	1.00±0.15	5140	0.017*
	DTI-FA	0.34±0.12	0.32±0.11	1815	0.279	0.31±0.10	0.36±0.11	4766	**0.002***
Kurtosis									
	DKI-AD	1.46±0.18	1.61±0.22	1013	**0.001***	1.45±0.19	1.58±0.26	4262	**<0.001***
	DKI-RD	0.84±0.17	0.96±0.20	1067	**0.002***	0.88±0.15	0.88±0.18	6495	0.779
	DKI-MD	1.05±0.15	1.18±0.18	947	**<0.001***	1.07±0.14	1.12±0.17	5431	0.069
	DKI-FA	0.35±0.11	0.33±0.11	1758	0.448	0.33±0.09	0.37±0.10	4706	**0.001***
	AK	0.63±0.09	0.62±0.10	1780	0.377	0.66±0.10	0.63±0.10	7525	0.021*
	RK	1.03±0.27	1.08±0.27	1444	0.308	1.01±0.21	1.11±0.25	4913	0.005*
	MK	0.82±0.13	0.84±0.16	1512	0.526	0.83±0.12	0.85±0.14	5798	0.274

	KFA	0.37±0.10	0.32±0.08	2093	0.008*	0.36±0.09	0.36±0.09	6635	0.578
	MKT	0.79±0.11	0.80±0.15	1559	0.713	0.80±0.12	0.81±0.13	6085	0.598
Fixel									
	FC	0.99±0.16	1.01±0.17	1584	0.821	0.98±0.14	1.00±0.15	5725	0.216
	FD	0.34±0.19	0.35±0.21	1595	0.869	0.31±0.15	0.38±0.18	4828	**0.003***
	FDC	0.35±0.23	0.36±0.23	1527	0.582	0.31±0.17	0.38±0.21	4799	**0.002***

Uncorrected p-values are provided. Correction for multiple comparisons will reduce the threshold for significance to p<0.0031 (p=0.05 divided by 16 parameters tested). Significant differences after correction for multiple comparisons are presented in bold.

Table 6: Comparison between black holes and other WML of SPMS

Diffusion parameter		BH (n=56)	WML (n=165)	Peri-lesion (n=221)	NAWM (n=221)	H	p-value
Tensor							
	DTI-AD	1.29±0.17	1.27±0.19	1.13±0.14	1.17±0.11	95203	**<0.001***
	DTI-RD	0.76±0.16	0.79±0.13	0.59±0.10	0.66±0.08	226801	**<0.001***
	DTI-MD	0.94±0.13	0.95±0.13	0.77±0.06	0.83±0.06	277613	**<0.001***
	DTI-FA	0.34±0.12	0.31±0.10	0.41±0.12	0.36±0.09	81612	**<0.001***
Kurtosis							
	DKI-AD	1.46±0.18	1.45±0.19	1.30±0.15	1.35±0.12	92492	**<0.001***
	DKI-RD	0.84±0.17	0.88±0.15	0.68±0.11	0.76±0.10	195398	**<0.001***
	DKI-MD	1.05±0.15	1.07±0.14	0.89±0.08	0.96±0.08	244984	**<0.001***

	DKI-FA	0.35±0.11	0.33±0.09	0.41±0.10	0.37±0.08	69349	**<0.001***
	AK	0.63±0.09	0.66±0.10	0.74±0.09	0.70±0.08	103430	**<0.001***
	RK	1.03±0.27	1.01±0.21	1.24±0.23	1.13±0.20	105194	**<0.001***
	MK	0.82±0.13	0.83±0.12	0.97±0.11	0.90±0.10	157814	**<0.001***
	KFA	0.37±0.10	0.36±0.09	0.43±0.09	0.40±0.07	62312	**<0.001***
	MKT	0.79±0.11	0.80±0.12	0.94±0.10	0.87±0.09	163078	**<0.001***
Fixel							
	FC	0.99±0.16	0.98±0.14	1.00±0.16	0.99±0.12	5704	0.127
	FD	0.34±0.19	0.31±0.15	0.41±0.15	0.37±0.12	54087	**<0.001***
	FDC	0.35±0.23	0.31±0.17	0.41±0.18	0.37±0.15	53639	**<0.001***

Uncorrected p-values are provided. Correction for multiple comparisons will reduce the threshold for significance to p<0.0031 (p=0.05 divided by 16 parameters tested). Significant differences after correction for multiple comparisons are presented in bold.

Table 7: Dunn's Post hoc test regarding SPMS

Diffusion analysis method	Diffusion parameter	Comparison	Z	Sig.	Adj. Sig.
Tensor					
	DTI-AD	BH vs. WML	-1.130	0.258	1.000
		BH vs. peri-lesion	-4.864	**<0.001***	**<0.001***
		BH vs. NAWM	-6.934	**<0.001***	**<0.001***
		WML vs. peri-lesion	-5.374	**<0.001***	**<0.001***
		WML vs. NAWM	-8.384	**<0.001***	**<0.001***
		Peri-lesion vs. NAWM	-3.256	**0.001***	**0.007***
	DTI-RD	BH vs. WML	-1.760	0.078	0.471
		BH vs. peri-lesion	-4.094	**<0.001***	**<0.001***
		BH vs. NAWM	-8.117	**<0.001***	**<0.001***
		WML vs. peri-lesion	-8.598	**<0.001***	**<0.001***
		WML vs. NAWM	-14.447	**<0.001***	**<0.001***
		Peri-lesion vs. NAWM	-6.326	**<0.001***	**<0.001***
	DTI-MD	BH vs. WML	-0.829	0.407	1.000
		BH vs. peri-lesion	-5.140	**<0.001***	**<0.001***
		BH vs. NAWM	-9.926	**<0.001***	**<0.001***

		WML vs. peri-lesion	-8.720	**<0.001***	**<0.001***
		WML vs. NAWM	-15.680	**<0.001***	**<0.001***
		Peri-lesion vs. NAWM	-7.527	**<0.001***	**<0.001***
	DTI-FA	BH vs. WML	-1.660	0.097	0.581
		BH vs. peri-lesion	-2.058	0.040*	0.238
		BH vs. NAWM	-4.331	**<0.001***	**<0.001***
		WML vs. peri-lesion	-5.488	**<0.001***	**<0.001***
		WML vs. NAWM	-8.794	**<0.001***	**<0.001***
		Peri-lesion vs. NAWM	-3.576	**<0.001***	**0.002***
Kurtosis					
	DKI-AD	BH vs. WML	-0.732	0.464	1.000
		BH vs. peri-lesion	-4.069	**<0.001***	**<0.001***
		BH vs. NAWM	-6.639	**<0.001***	**<0.001***
		WML vs. peri-lesion	-4.816	**<0.001***	**<0.001***
		WML vs. NAWM	-8.553	**<0.001***	**<0.001***
		Peri-lesion vs. NAWM	-4.041	**<0.001***	**<0.001***
	DKI-RD	BH vs. WML	-1.843	0.065	0.392
		BH vs. peri-lesion	-3.010	**<0.001***	**<0.001***
		BH vs. NAWM	-7.388	**0.003***	**0.016***
		WML vs. peri-lesion	-7.147	**<0.001***	**<0.001***
		WML vs. NAWM	-13.514	**<0.001***	**<0.001***
		Peri-lesion vs. NAWM	-6.886	**<0.001***	**<0.001***
	DKI-MD	BH vs. WML	-0.992	0.321	1.000
		BH vs. peri-lesion	-3.952	**<0.001***	**<0.001***
		BH vs. NAWM	-9.163	**<0.001***	**<0.001***
		WML vs. peri-lesion	-7.238	**<0.001***	**<0.001***
		WML vs. NAWM	-14.815	**<0.001***	**<0.001***
		Peri-lesion vs. NAWM	-8.195	**<0.001***	**<0.001***
	DKI-FA	BH vs. WML	-1.443	0.149	0.893
		BH vs. peri-lesion	-1.772	**<0.001***	**<0.001***
		BH vs. NAWM	-4.091	0.076	0.458
		WML vs. peri-lesion	-4.746	**<0.001***	**<0.001***
		WML vs. NAWM	-8.118	**<0.001***	**<0.001***
		Peri-lesion vs. NAWM	-3.647	**<0.001***	**<0.001***

	AK	BH vs. WML	-2.662	**0.008***	**0.047***
		BH vs. peri-lesion	-5.494	**<0.001***	**<0.001***
		BH vs. NAWM	-8.284	**<0.001***	**<0.001***
		WML vs. peri-lesion	-3.988	**<0.001***	**<0.001***
		WML vs. NAWM	-8.045	**<0.001***	**<0.001***
		Peri-lesion vs. NAWM	-4.388	**<0.001***	**<0.001***
	RK	BH vs. WML	-0.244	0.807	1.000
		BH vs. peri-lesion	-3.232	**0.001***	**0.007***
		BH vs. NAWM	-6.329	**<0.001***	**<0.001***
		WML vs. peri-lesion	-5.067	**<0.001***	**<0.001***
		WML vs. NAWM	-9.570	**<0.001***	**<0.001***
		Peri-lesion vs. NAWM	-4.870	**<0.001***	**<0.001***
	MK	BH vs. WML	-0.571	0.568	1.000
		BH vs. peri-lesion	-4.039	**<0.001***	**<0.001***
		BH vs. NAWM	-8.408	**<0.001***	**<0.001***
		WML vs. peri-lesion	-5.015	**<0.001***	**<0.001***
		WML vs. NAWM	-11.368	**<0.001***	**<0.001***
		Peri-lesion vs. NAWM	-6.871	**<0.001***	**<0.001***
	KFA	BH vs. WML	-0.040	0.968	1.000
		BH vs. peri-lesion	-2.746	**0.006***	**0.036***
		BH vs. NAWM	-4.978	**<0.001***	**<0.001***
		WML vs. peri-lesion	-4.054	**<0.001***	**<0.001***
		WML vs. NAWM	-7.300	**<0.001***	**<0.001***
		Peri-lesion vs. NAWM	-3.511	**<0.001***	**0.003***
	MKT	BH vs. WML	-0.918	0.359	1.000
		BH vs. peri-lesion	-4.394	**<0.001***	**<0.001***
		BH vs. NAWM	-8.797	**<0.001***	**<0.001***
		WML vs. peri-lesion	-5.009	**<0.001***	**<0.001***
		WML vs. NAWM	-11.412	**<0.001***	**<0.001***
		Peri-lesion vs. NAWM	-6.925	**<0.001***	**<0.001***
Fixel					
	FBA-FC	BH vs. WML	n.a.	n.a.	n.a.
		BH vs. peri-lesion			
		BH vs. NAWM			

		WML vs. peri-lesion<br>WML vs. NAWM<br>Peri-lesion vs. NAWM			
	FBA-FD	BH vs. WML<br>BH vs. peri-lesion<br>BH vs. NAWM<br>WML vs. peri-lesion<br>WML vs. NAWM<br>Peri-lesion vs. NAWM	-1.237<br>-2.141<br>-3.551<br>-4.973<br>-7.023<br>-2.217	0.216<br>0.032*<br>**<0.001***<br>**<0.001***<br>**<0.001***<br>0.027*	1.000<br>0.194<br>**0.002***<br>**<0.001***<br>**<0.001***<br>0.160
	FBA-FDC	BH vs. WML<br>BH vs. peri-lesion<br>BH vs. NAWM<br>WML vs. peri-lesion<br>WML vs. NAWM<br>Peri-lesion vs. NAWM	-0.875<br>-2.363<br>-3.862<br>-4.751<br>-6.931<br>-2.358	0.382<br>0.018*<br>**<0.001***<br>**<0.001***<br>**<0.001***<br>0.018*	1.000<br>0.109<br>**0.001***<br>**<0.001***<br>**<0.001***<br>0.110

Sign. are uncorrected p-values. Adj. sign. are Bonferroni corrected p-values

Table 8: Comparison between black holes and other WML of PPMS

Diffusion parameter		BH (n=58)	WML (n=77)	Peri-lesion (n=135)	NAWM (n=135)	H	p-value
Tensor							
	DTI-AD	1.44±0.22	1.41±0.23	1.19±0.18	1.27±0.14	86743	**<0.001***
	DTI-RD	0.88±0.18	0.80±0.17	0.62±0.11	0.69±0.10	131798	**<0.001***
	DTI-MD	1.07±0.16	1.00±0.15	0.81±0.08	0.89±0.08	175604	**<0.001***
	DTI-FA	0.32±0.11	0.36±0.11	0.41±0.13	0.38±0.09	33239	**<0.001***
Kurtosis							
	DKI-AD	1.61±0.22	1.58±0.26	1.37±0.20	1.46±0.15	75015	**<0.001***
	DKI-RD	0.96±0.20	0.88±0.18	0.70±0.12	0.79±0.11	110068	**<0.001***

	DKI-MD	1.18±0.18	1.12±0.17	0.92±0.09	1.01±0.09	149390	**<0.001***
	DKI-FA	0.33±0.11	0.37±0.10	0.41±0.11	0.39±0.08	26897	**<0.001***
	AK	0.62±0.10	0.63±0.10	0.75±0.10	0.69±0.07	97271	**<0.001***
	RK	1.08±0.27	1.11±0.25	1.43±0.29	1.25±0.20	97661	**<0.001***
	MK	0.84±0.16	0.85±0.14	1.06±0.13	0.95±0.12	126443	**<0.001***
	KFA	0.32±0.08	0.36±0.09	0.40±0.10	0.37±0.08	29859	**<0.001***
	MKT	0.80±0.15	0.81±0.13	1.00±0.10	0.90±0.10	132457	**<0.001***
Fixel							
	FC	1.01±0.17	1.00±0.15	1.08±0.18	1.02±0.15	14122	**0.003***
	FD	0.35±0.21	0.38±0.18	0.44±0.17	0.42±0.13	21438	**<0.001***
	FDC	0.36±0.23	0.38±0.21	0.47±0.21	0.43±0.16	30406	**<0.001***

Uncorrected p-values are provided. Correction for multiple comparisons will reduce the threshold for significance to p<0.0031 (p=0.05 divided by 16 parameters tested). Significant differences after correction for multiple comparisons are presented in bold.

Table 9: Dunn's post-hoc test regarding PPMS

Diffusion analysis method	Diffusion parameter	Comparison	Z	Sig.	Adj. Sig.
Tensor					
	DTI-AD	BH vs. WML	-0.737	0.461	1.000
		BH vs. peri-lesion	-4.290	**<0.001***	**<0.001***
		BH vs. NAWM	-7.659	**<0.001***	**<0.001***
		WML vs. peri-lesion	-3.819	**<0.001***	**0.001***
		WML vs. NAWM	-7.522	**<0.001***	**<0.001***
		Peri-lesion vs. NAWM	-4.345	**<0.001***	**<0.001***
	DTI-RD	BH vs. WML	-1.928	0.054	0.324
		BH vs. peri-lesion	-6.149	**<0.001***	**<0.001***
		BH vs. NAWM	-9.968	**<0.001***	**<0.001***
		WML vs. peri-lesion	-4.413	**<0.001***	**<0.001***
		WML vs. NAWM	-8.612	**<0.001***	**<0.001***
		Peri-lesion vs. NAWM	-4.926	**<0.001***	**<0.001***
	DTI-MD	BH vs. WML	-1.696	0.090	0.539

		BH vs. peri-lesion	-6.267	**<0.001***	**<0.001***
		BH vs. NAWM	-11.260	**<0.001***	**<0.001***
		WML vs. peri-lesion	-4.825	**<0.001***	**<0.001***
		WML vs. NAWM	-10.314	**<0.001***	**<0.001***
		Peri-lesion vs. NAWM	-6.439	**<0.001***	**<0.001***
	DTI-FA	BH vs. WML	-2.497	0.013*	0.075
		BH vs. peri-lesion	-4.490	**<0.001***	**<0.001***
		BH vs. NAWM	-5.444	**<0.001***	**<0.001***
		WML vs. peri-lesion	-1.897	0.058	0.347
		WML vs. NAWM	-2.945	**0.003***	**0.019***
		Peri-lesion vs. NAWM	-1.230	0.219	1.000
Kurtosis					
	DKI-AD	BH vs. WML	-0.958	0.338	1.000
		BH vs. peri-lesion	-3.948	**<0.001***	**<0.001***
		BH vs. NAWM	-7.279	**<0.001***	**<0.001***
		WML vs. peri-lesion	-3.175	**0.002***	**0.009***
		WML vs. NAWM	-6.836	**<0.001***	**<0.001***
		Peri-lesion vs. NAWM	-4.295	**<0.001***	**<0.001***
	DKI-RD	BH vs. WML	-2.001	0.045*	0.272
		BH vs. peri-lesion	-4.935	**<0.001***	**<0.001***
		BH vs. NAWM	-9.240	**<0.001***	**<0.001***
		WML vs. peri-lesion	-2.990	**0.003***	**0.017***
		WML vs. NAWM	-7.722	**<0.001***	**<0.001***
		Peri-lesion vs. NAWM	-5.552	**<0.001***	**<0.001***
	DKI-MD	BH vs. WML	-1.768	0.077	0.462
		BH vs. peri-lesion	-5.036	**<0.001***	**<0.001***
		BH vs. NAWM	-10.453	**<0.001***	**<0.001***
		WML vs. peri-lesion	-3.384	**0.001***	**0.004***
		WML vs. NAWM	-9.339	**<0.001***	**<0.001***
		Peri-lesion vs. NAWM	-6.987	**<0.001***	**<0.001***
	DKI-FA	BH vs. WML	-2.314	0.021*	0.124
		BH vs. peri-lesion	-3.707	**<0.001***	**<0.001***
		BH vs. NAWM	-5.019	**<0.001***	**0.001***
		WML vs. peri-lesion	-1.258	0.208	1.000

		WML vs. NAWM	-2.701	**0.007***	**0.042***
		Peri-lesion vs. NAWM	-1.693	0.090	0.543
	AK	BH vs. WML	-0.477	0.633	1.000
		BH vs. peri-lesion	-3.873	**<0.001***	**0.001***
		BH vs. NAWM	-7.934	**<0.001***	**<0.001***
		WML vs. peri-lesion	-3.676	**<0.001***	**0.001***
		WML vs. NAWM	-8.141	**<0.001***	**<0.001***
		Peri-lesion vs. NAWM	-5.238	**<0.001***	**<0.001***
	RK	BH vs. WML	-0.646	0.518	1.000
		BH vs. peri-lesion	-4.204	**<0.001***	**<0.001***
		BH vs. NAWM	-8.053	**<0.001***	**<0.001***
		WML vs. peri-lesion	-3.836	**<0.001***	**0.001***
		WML vs. NAWM	-8.067	**<0.001***	**<0.001***
		Peri-lesion vs. NAWM	-4.965	**<0.001***	**<0.001***
	MK	BH vs. WML	-0.310	0.757	1.000
		BH vs. peri-lesion	-3.967	**<0.001***	**<0.001***
		BH vs. NAWM	-8.885	**<0.001***	**<0.001***
		WML vs. peri-lesion	-3.984	**<0.001***	**<0.001***
		WML vs. NAWM	-9.390	**<0.001***	**<0.001***
		Peri-lesion vs. NAWM	-6.343	**<0.001***	**<0.001***
	KFA	BH vs. WML	-2.539	0.011*	0.067
		BH vs. peri-lesion	-3.955	**<0.001***	**<0.001***
		BH vs. NAWM	-5.311	**<0.001***	**<0.001***
		WML vs. peri-lesion	-1.257	0.209	1.000
		WML vs. NAWM	-2.747	**0.006***	**0.036***
		Peri-lesion vs. NAWM	-1.749	0.080	0.482
	MKT	BH vs. WML	-0.358	0.720	1.000
		BH vs. peri-lesion	-4.034	**<0.001***	**<0.001***
		BH vs. NAWM	-9.114	**<0.001***	**<0.001***
		WML vs. peri-lesion	-3.998	**<0.001***	**<0.001***
		WML vs. NAWM	-9.584	**<0.001***	**<0.001***
		Peri-lesion vs. NAWM	-6.553	**<0.001***	**<0.001***
Fixel					
	FBA-FC	BH vs. WML	-0.406	0.684	1.000

		BH vs. peri-lesion	-1.298	0.194	1.000
		BH vs. NAWM	-3.118	**0.002***	**0.011***
		WML vs. peri-lesion	-0.932	0.351	1.000
		WML vs. NAWM	-2.933	**0.003***	**0.020***
		Peri-lesion vs. NAWM	-2.348	0.019*	0.113
	FBA-FD	BH vs. WML	-1.360	0.174	1.000
		BH vs. peri-lesion	-3.841	**<0.001***	**0.001***
		BH vs. NAWM	-3.820	**<0.001***	**0.001***
		WML vs. peri-lesion	-2.567	0.010*	0.066
		WML vs. NAWM	-2.544	0.011*	0.061
		Peri-lesion vs. NAWM	-0.028	0.978	1.000
	FBA-FDC	BH vs. WML	-1.183	0.237	1.000
		BH vs. peri-lesion	-3.819	**<0.001***	**0.001***
		BH vs. NAWM	-4.732	**<0.001***	**<0.001***
		WML vs. peri-lesion	-2.758	**0.006***	**0.035***
		WML vs. NAWM	-3.762	**<0.001***	**0.001***
		Peri-lesion vs. NAWM	-1.178	0.239	1.000

Sign. are uncorrected p-values. Adj. sign. are Bonferroni corrected p-values

Table 10: Diffusion parameter evaluation in MS lesions. Data are shown as average (± standard deviation)

Diffusion parameter		Lesion	NAWM	U	T (df)	Sig (p-value)
Tensor						
	DTI-AD	1.33 (±0.21)	1.16 (±0.16)	99166.5		**<0.001***
	DTI-RD	0.80 (±0.16)	0.60 (±0.10)	109954.5		**<0.001***
	DTI-MD	0.98 (±0.15)	0.79 (±0.07)	114522.5		**<0.001***
	DTI-FA	0.33 (±0.11)	0.41 (±0.12)	37190		**<0.001***
Kurtosis						
	DKI-AD	1.51 (±0.22)	1.33 (±0.17)	95288		**<0.001***
	DKI-RD	0.89 (±0.17)	0.69 (±0.11)	106525		**<0.001***
	DKI-MD	1.09 (±0.16)	0.90 (±0.08)	111717		**<0.001***
	DKI-FA	0.34 (±0.10)	0.41 (±0.11)	102679		**<0.001***
	AK	0.64 (±0.10)	0.74 (±0.09)		-13.9 (701)	**<0.001***

	RK	1.05 (±0.24)	1.32 (±0.27)	27458		**<0.001***
	MK	0.83 (±0.14)	1.01 (±0.12)	20978		**<0.001***
	KFA	0.35 (±0.09)	0.42 (±0.10)	39714		**<0.001***
	MKT	0.80 (±0.12)	0.96 (±0.10)		-18.5 (688)	**<0.001***
Fixel						
	FC	0.99 (±0.15)	1.03 (±0.17)	52747		**<0.001***
	FD	0.33 (±0.18)	0.42 (±0.16)	42103		**<0.001***
	FDC	0.34 (±0.20)	0.44 (±0.19)	40372		**<0.001***

Uncorrected p-values are provided. Correction for multiple comparisons will reduce the threshold for significance to p<0.0031 (p=0.05 divided by 16 parameters tested). Significant differences after correction for multiple comparisons are presented in bold.

Table 11: Diffusion parameter evaluation in peri-lesion white matter. Data are shown as average (± standard deviation)

Diffusion analysis method	Diffusion parameter	Peri-lesion	NAWM	U	T (df)	Sig (p-value)
Tensor						
	DTI-AD	1.21 (±0.13)	1.16 (±0.16)	79949		**<0.001***
	DTI-RD	0.67 (±0.09)	0.60 (±0.10)	89592.5		**<0.001***
	DTI-MD	0.85 (±0.07)	0.79 (±0.070)	95701.5		**<0.001***
	DTI-FA	0.38 (±0.10)	0.41 (±0.12)	52047.5		**<0.001***
Kurtosis						
	DKI-AD	1.39 (±0.14)	1.33 (±0.17)	81517.5		**<0.001***
	DKI-RD	0.78 (±0.10)	0.69 (±0.11)		10.3 (710)	**<0.001***
	DKI-MD	0.98 (±0.09)	0.90 (±0.08)	96926		**<0.001***
	DKI-FA	0.38 (±0.081)	0.41 (±0.11)	51296.5		**<0.001***
	AK	0.79 (±0.08)	0.74 (±0.09)		-7.542 (686)	**<0.001***
	RK	1.17 (±0.21)	1.32 (±0.27)	43224.5		**<0.001***
	MK	0.92 (±0.11)	1.01 (±0.12)	35240.5		**<0.001***
	KFA	0.39 (±0.08)	0.42 (±0.10)	51776		**<0.001***

	MKT	0.88 (±0.10)	0.96 (±0.10)		-10.6 (710)	**<0.001***
Fixel						
	FBA-FC	1.01 (±0.13)	1.03 (±0.17)	55947.5		0.007*
	FBA-FD	0.39 (±0.12)	0.42 (±0.16)	58029		0.052
	FBA-FDC	0.39 (±0.15)	0.44 (±0.19)	55733		0.005*

Uncorrected p-values are provided. Correction for multiple comparisons will reduce the threshold for significance to $p<0.0031$ ($p=0.05$ divided by 16 parameters tested). Significant differences after correction for multiple comparisons are presented in bold.

Table 12: Results of the statistical analysis of the differences in diffusion parameters between MS lesion and peri-lesion white matter. Data are shown as average (± standard deviation)

Diffusion analysis method	Diffusion parameter	Lesion	Peri-lesion	U	T (df)	Sig (p-value)
Tensor						
	DTI-AD	1.33 (±0.21)	1.21 (±0.13)	40428		**<0.001***
	DTI-RD	0.80 (±0.16)	0.67 (±0.09)		13.613 (568.626)	**<0.001***
	DTI-MD	0.98 (±0.15)	0.85 (±0.07)	27368		**<0.001***
	DTI-FA	0.33 (±0.11)	0.38 (±0.10)	82426.5		**<0.001***
Kurtosis						
	DKI-AD	1.51 (±0.22)	1.39 (±0.14)	43174		**<0.001***
	DKI-RD	0.89 (±0.17)	0.78 (±0.10)	36695.5		**<0.001***
	DKI-MD	1.09 (±0.16)	0.98 (±0.09)	34650		**<0.001***
	DKI-FA	0.34 (±0.10)	0.38 (±0.081)	79372		**<0.001***
	AK	0.64 (±0.10)	0.79 (±0.08)	85094		**<0.001***

	RK	1.05 (±0.24)	1.17 (±0.21)		-7.996 (654.060)	**<0.001***
	MK	0.83 (±0.14)	0.92 (±0.11)		-9.000 (679.535)	**<0.001***
	KFA	0.35 (±0.09)	0.39 (±0.08)	78687		**<0.001***
	MKT	0.80 (±0.12)	0.88 (±0.10)		-9.343 (673.138)	**<0.001***
Fixel						
	FBA-FC	0.99 (±0.15)	1.01 (±0.13)	67182		0.165
	FBA-FD	0.33 (±0.18)	0.39 (±0.12)	80949		**<0.001***
	FBA-FDC	0.34 (±0.20)	0.39 (±0.15)	81038		**<0.001***

Uncorrected p-values are provided. Correction for multiple comparisons will reduce the threshold for significance to $p<0.0031$ ($p=0.05$ divided by 16 parameters tested). Significant differences after correction for multiple comparisons are presented in bold.
